# Assessment of Serum Tumor Markers for Predicting Ocular Metastasis in Lung Adenocarcinoma: A Retrospective Study

**DOI:** 10.1155/2020/2102158

**Published:** 2020-06-25

**Authors:** Wen-Qing Shi, Wen-Feng Liu, Biao Li, Qi Lin, Qing-Hai Li, Yu-Qing Zhang, Qing Yuan, Rong-Bin Liang, Qian-Min Ge, Yi Shao

**Affiliations:** ^1^Department of Ophthalmology, The First Affiliated Hospital of Nanchang University, Jiangxi Province Clinical Ophthalmology Institute, Nanchang, 330006 Jiangxi, China; ^2^Department of Hepatic Oncology, Zhongshan Hospital, Fudan University, Shanghai 200032, China

## Abstract

The purpose of this study was to detect clinical variations between lung adenocarcinoma patients with and without ocular metastasis (OM) to identify risk factors for OM and assess the diagnostic values. We included 1153 patients with lung adenocarcinoma in this study. Independent *t*-tests and chi-square tests were used to compare patients' clinical characteristics. Statistically significant parameters were analyzed by binary logistic regression to detect risk factors of OM. The results showed that the OM group had increased alpha-fetoprotein (AFP), carcinoembryonic antigen (CEA), cytokeratin fragment 19 (CYFRA 21-1), carbohydrate antigen- (CA-) 125, CA-153, and total prostate-specific antigen (TPSA) compared with the NOM group. CYFRA21-1 is the most useful biomarker for detecting OM in this population.

## 1. Introduction

Lung cancer is a common malignant tumor worldwide. The incidence and mortality of lung cancer rank first among all cancer types in males, accounting for 17% of all new cancer cases and 23% of cancer-related deaths [1]. The female incidence of lung cancer has continually increased in recent years, and it is now the second most common type of cancer in women [2]. Causes of lung cancer include air pollution and exposure to occupational and environmental carcinogens [3, 4]. Normal epithelial cells experience multiple genetic insults and eventually undergo abnormal growth and develop invasive behaviors. Lung cancer can be divided into small cell and nonsmall cell according to the histological type. The latter group includes squamous cell cancer, adenocarcinoma, and large cell cancer. Most lung adenocarcinomas originate from the bronchial mucosal epithelium, while a few develop from the mucous glands of bronchia. However, the prognosis of lung adenocarcinoma is poor [5, 6].

The eye is a rare metastatic site for malignant tumors. Ocular metastasis (OM) is the most often secondary to, for example, breast, lung, kidney, or prostate cancer [7, 8]. Lung adenocarcinoma is usually diagnosed at an advanced stage, and patients often have distant metastases [9]. The most common sites are the brain, bone, liver, and adrenal gland [10, 11]. In the early stage, OM may have not been accompanied by ophthalmic symptoms. Over time, patients with OM may have blurred vision, pain, visual field defects, flashes, and diplopia [12]. OM can also result in pupil deformation and secondary glaucoma [13]. These symptoms seriously affect the quality of life and shorten the survival time of patients with lung adenocarcinoma. Some studies have also reported asymptomatic cases of choroidal metastasis [14, 15]. Given the shortcomings of conventional computed tomography (CT) and magnetic resonance imaging (MRI) in detecting eye metastases, a simple and economical technique for predicting eye metastases would be clinically useful.

Tumor markers are widely used for clinical screening. Detection of markers in the blood, body fluid, or cells can facilitate diagnosis, help clarify the pathogenesis, and inform prognosis. Many serum tumor markers are associated with lung cancer. For example, Ma et al. [16] found increased levels of carcinoembryonic antigen (CEA), carbohydrate antigen- (CA-) 125, and cytokeratin fragment 19 (CYFRA21-1) in lung cancer patients, which could be used for diagnostic purposes. Previous studies have also shown that CEA, CYFRA21-1, and CA-125 are associated with worse prognosis in non-small-cell lung cancer [17].

Tumor markers may be effective markers for predicting metastasis. However, it is not clear whether there are differences in tumor markers used in the diagnosis of OM and NOM patients with lung adenocarcinoma. In this study, we collected medical records of lung adenocarcinoma patients treated at the First Affiliated Hospital of Nanchang University and retrospectively analyzed the diagnostic value of several tumor markers, which might provide a medical basis for predicting OM in this population.

## 2. Materials and Methods

### 2.1. Study Design

All patients volunteered to participate. This study was approved by the Medical Research Ethics Committee of the First Affiliated Hospital of Nanchang University. The patients were diagnosed with lung adenocarcinoma between October 2001 and February 2017 based on pathological sections obtained by surgical excision or biopsy. OM was diagnosed by CT and MRI. Patients with primary ocular malignancies, benign ocular tumors, and secondary lung cancer were excluded.

### 2.2. Data Collection

We collected clinical data including age, sex, and treatment from the patients' medical records. Some tumor markers were also detected, including calcium, hemoglobin (HB), alkaline phosphatase (ALP), alpha-fetoprotein (AFP), CEA, neuron-specific enolase (NSE), CYFRA 21-1, CA-125, CA-153, CA-199, and total prostate-specific antigen (TPSA). All data were collected when the patients were diagnosed with lung adenocarcinoma.

### 2.3. Statistical Analysis

We performed independent *t*-tests (age, tumor markers) and chi-square tests (sex) to compare the OM and NOM groups. Binary logistic regression models were then applied to identify independent risk factors for OM. We then constructed receiver operating characteristic (ROC) curves and areas under the curves (AUCs). Excel 2010 software (Microsoft, Washington, USA) was used to calculate the cutoff value, sensitivity, and specificity of each biomarker. Differences were considered significant at *p* < 0.05. All statistical analyses were performed using SPSS 20.0 software (SPSS, IBM, USA) and Excel 2010 software.

## 3. Results

### 3.1. Demographics and Clinical Characteristics

**Figure 1:**
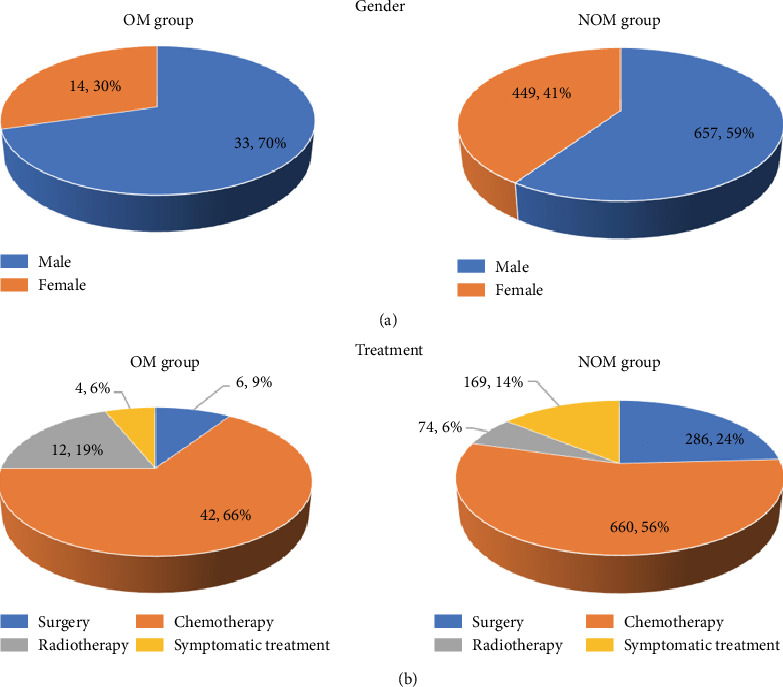
The clinical characteristics of patients with lung adenocarcinoma. OM: ocular metastasis; NOM: nonocular metastasis.

We recruited 1153 patients (47 OM, 1106 NOM). The mean ages of the OM and NOM groups were 59.0 ± 1.5 and 59.2 ± 0.3 years, respectively. No significant differences were noted in sex or age between the OM and NOM groups (*p* > 0.05). Full details are shown in Table 1 and Figure 1. Other metastatic sites of the OM group were the lung (38.3%), lymph node (59.6%), brain (97.9%), bone (57.4%), liver (17.0%), peritoneum (4.3%), and pleura (6.4%), while metastatic sites of the NOM group were the lung (32.5%), lymph node (61.7%), brain (16.0%), bone (35.2%), liver (15.8%), peritoneum (12.4%), and pleura (0.5%). See more details in Table 2.

### 3.2. Differences in the Clinical Features and Risk Factors of Patients with OM

There were no significant differences in the levels of calcium, HB, ALP, NSE, or CA-199 between the OM and NOM groups (*p* > 0.05). However, we found higher levels of AFP, CEA, CYFRA 21-1, CA-125, CA-153, and TPSA in the OM group (*p* < 0.05). Detailed results are listed in Table 3. Binary logistic regression modeling identified CYFRA 21-1, CA-125, and CA-153 as independent risk factors of OM in patients with lung adenocarcinoma. Details are shown in Table 4.

### 3.3. Cutoff Values, AUCs, Sensitivities, and Specificities of Biomarkers for OM Diagnosis

**Figure 2:**
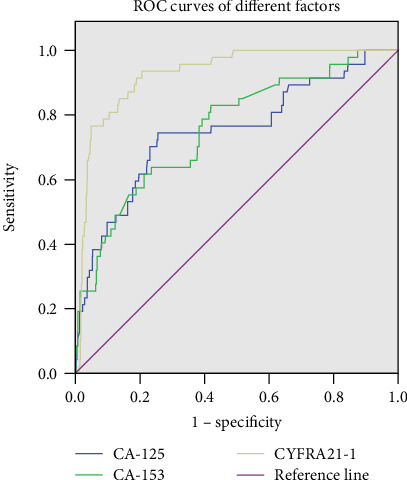
The ROC curves of risk factor for detecting OM in lung adenocarcinoma. ROC curves of CYFRA21-1, CA-123, and CA-153 as a single risk factor of OM. ROC: receiver operating characteristic; OM: ocular metastasis.

**Figure 3:**
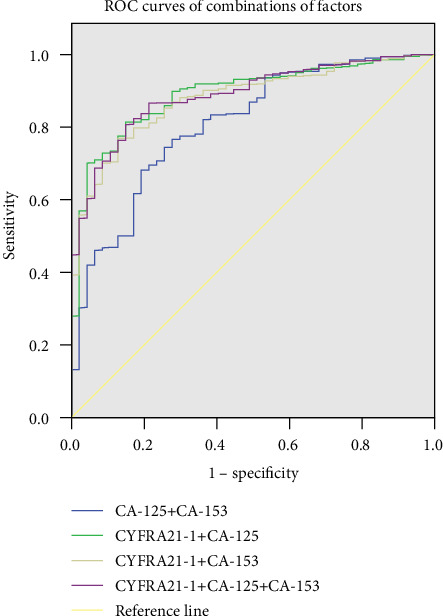
The ROC curves of combinations of different combinations of risk factors for detecting OM in lung adenocarcinoma patients and the ROC curves of CYFRA 21-1+CA-125, CYFRA 21-1+CA-153, CA-125+CA-153, and CYFRA 21-1+CA-125+CA-153. ROC: receiver operating characteristic; OM: ocular metastasis.

ROC curves (Figure 2) showed that the AUCs for CYFRA 21-1, CA-125, and CA-153 were 0.928, 0.749, and 0.758, respectively. The cutoff values of CYFRA 21-1, CA-125, and CA-153 were 6.785 ng/mL, 66.295 U/mL, and 13.005 U/mL, respectively. The sensitivities and specificities of diagnosing OM by CYFRA21-1 were 93.6% and 79.4%, by CA-125, they were 74.5% and 74.4%, and by CA-153, they were 83.0% and 58.0%, respectively (Table 5). Combinations of the three factors were also calculated, and Figure 3 shows the ROC curves for CYFRA 21-1+CA-125, CYFRA 21-1+CA-153, CA-125+CA-153, and CYFRA 21-1+CA-125+CA-153. The highest AUC value was for CYFRA 21-1.

## 4. Discussion

Lung adenocarcinoma has become one of the most common diseases with high morbidity and mortality. OM is rare in lung adenocarcinoma patients, but the clinical symptoms are serious. OM seriously affects the quality of life of patients and may suggest poor prognosis, so early prediction is of great significance.

OM that appears in lung cancer patients is caused by hematogenous dissemination of tumor cells [18]. In a study including 229 eyes of 194 patients with uveal metastasis [19], tumors were located in the choroid (88%), iris (10%), and ciliary body (2%), with bilateral uveal (18%). The choroid is supplied by several thick, short posterior ciliary arteries in the rear of the eye, and there are extensive anastomotic branches between the choroidal vessels. The lumen is larger, and blood flow is abundant but slow [20]. For these reasons, choroidal metastasis is the most common clinical presentation. Metastasis to the orbit, eyelid, conjunctiva, retina, and optic nerve has also been reported [21, 22]. Interestingly, OM is more common in the left eye. This is because the left common carotid artery branches directly from the aortic arch, allowing tumor emboli to easily enter the left eye, whereas right eye access requires bypassing the anonymous artery.

Traditional histopathological and immunohistochemical examinations and cytogenetic analyses are tedious and time-consuming. Once a diagnosis of lung cancer is made, physicians can choose multimodal treatment including surgery, radiotherapy, and chemotherapy. Tumor markers mainly exist in serum and can be detected by immunological, biological, and chemical methods [23]. Compared with CT and MRI, they are economical and convenient. Most importantly, tumor markers have a predictive value.  Table 6 shows the risk factors for distant metastasis of lung cancer reported in previous studies.

After analyzing clinical data from 1153 patients with lung adenocarcinoma, we found that the concentrations of AFP, CEA, CYFRA 21-1, CA-125, CA-153, and TPSA were significantly elevated in patients with OM. However, AFP is associated with liver metastasis [24], and TPSA is associated with benign prostatic hyperplasia, prostate cancer, and bone metastasis [25, 26]. We therefore excluded AFP and TPSA from the tumor markers under consideration as OM markers. According to the binary logistic regression result, CYFRA 21-1, CA-125, and CA-153 were identified as possible independent risk factors for OM in patients with lung adenocarcinoma. CYFRA 21-1 is a fragment of cytokeratin-19 (an epithelial cell filament) produced during cancer cell differentiation. This marker has been associated with lung cancer, colorectal tumors, and bladder cancer [27–29], as well as with distant metastasis in patients with lung cancer [30]. In a retrospective study, Chen et al. [31] found that CYFRA 21-1 was an independent risk factor for predicting lymph node metastasis in lung cancer, with a sensitivity and specificity of 0.375 and 0.85, respectively. CA-125, also known as tumor antigen 125, is a high molecular weight glycoprotein produced by the *MUC16* gene that is expressed on the surface of epithelial ovarian tumors and mesothelium-derived cells. CA-125 has been widely used to diagnose and assess progression and treatment effects for multiple types of cancer [32, 33] CA-125 levels were also found to be useful for predicting lymph node metastasis in patients with ovarian cancer [34]. CA-153 was first identified on breast cancer cell membranes with a relative molecular weight of 4000. Abnormal CA-153 levels may also be measured in patients with many types of tumors including lung cancer [35]. It can be used to predict bone metastasis in breast cancer patients [36]. Based on the analyses of CYFRA 21-1, CA-125, and CA-153 levels and the high AUCs of the three biomarkers, we concluded that they are independent risk factors for OM in patients with lung adenocarcinoma.

The cutoff values showed that CYFRA 21-1 > 6.785 ng/mL, CA-125 > 66.295 U/mL, and CA-125 > 13.005 U/mL were associated with OM in patients with lung adenocarcinoma. The highest AUC was observed for CYFRA 21-1, suggesting its superior diagnostic value in predicting OM. We also analyzed diagnostic accuracy rates for different combinations of these risk factors. However, the AUC values of these combinations were lower than that of CYFRA 21-1.

Some limitations of this study should be mentioned. First, the group of patients with OM was small, and these findings should be replicated in a larger cohort. Secondly, the participants were from the same hospital, which may limit the generalizability of our results.

Based on the analysis of 1153 patients with lung adenocarcinoma, we conclude that the serum concentrations of CYFRA 21-1, CA-125, and CA-153 are independent risk factors for OM. Among these, CYFRA 21-1 has the highest accuracy in predicting OM.

## Figures and Tables

**Table 1:** The clinical characteristics of patients with lung adenocarcinoma.

Patient characteristics	OM group	NOM group	*p* value
	(*n* = 47)	(*n* = 1106)	
Gender^a^			
Male	33	657	0.139
Female	14	449	
Age^b^			
Mean	59.0 ± 1.5	59.2 ± 0.3	0.927
Treatment			
Surgery	6	286	
Chemotherapy	42	660	
Radiotherapy	12	74	
Symptomatic treatment	4	169	

^a^A chi-square test was applied. ^b^Student's *t*-test was applied. *p* < 0.05 was thought to be statistical significance. OM: ocular metastasis; NOM: nonocular metastasis.

**Table 2:** Other metastatic sites of the OM and NOM groups.

Sites	OM	NOM
Lung	18 (38.3%)	359 (32.5%)
Lymph node	28 (59.6%)	682 (61.7%)
Brain	46 (97.9%)	177 (16.0%)
Bone	27 (57.4%)	389 (35.2%)
Liver	8 (17.0%)	175 (15.8%)
Peritoneum	2 (4.3%)	137 (12.4%)
Pleura	3 (6.4%)	6 (0.5%)

OM: ocular metastasis; NOM: nonocular metastasis.

**Table 3:** Differences of tumor markers between lung adenocarcinoma patients with and without OM.

Tumor markers	OM group	NOM group	*t*	*p* value
Calcium (mmol/L)	2.28 ± 0.03	2.25 ± 0.01	0.711	0.477
HB (g/L)	114.62 ± 3.26	119.08 ± 0.56	-1.594	0.111
ALP (U/L)	117.40 ± 8.74	99.29 ± 3.38	1.099	0.272
AFP (ng/mL)	3.15 ± 0.30	1.86 ± 0.04	4.326	<0.001
CEA (ng/mL)	239.85 ± 82.71	62.67 ± 9.02	2.130	0.038
NSE (*μ*g/L)	26.29 ± 2.24	23.90 ± 1.06	0.460	0.645
CYFRA21-1 (ng/mL)	41.02 ± 5.02	9.81 ± 1.05	6.086	<0.001
CA-125 (U/mL)	413.05 ± 84.25	86.20 ± 6.29	3.869	<0.001
CA-153 (U/mL)	89.24 ± 18.04	22.83 ± 1.08	3.675	0.001
CA-199 (U/mL)	158.59 ± 50.57	69.96 ± 18.84	0.963	0.336
TPSA (ng/L)	4.28 ± 0.33	1.59 ± 0.10	7.759	<0.001

Independent sample *t*-test was applied. *p* < 0.05 represented statistical significance. OM: ocular metastasis; NOM: nonocular metastasis.

**Table 4:** Risk factors of OM in patients with lung adenocarcinoma.

Factors	B	Exp (*B*)	OR (95% CI)	*p* value
CEA	0.000	1.000	0.999-1.000	0.671
CYFRA21-1	-0.009	0.991	0.987-0.995	<0.001
CA-125	-0.001	0.999	0.998-1.000	0.005
CA-153	-0.008	0.992	0.987-0.996	<0.001

Binary logistic analysis was applied. *p* < 0.05 represented statistical significance. *B*: coefficient of regression; OR: odds ratio; CI: confidence interval; OM: ocular metastasis.

**Table 5:** The cutoff value, sensitivity, specificity, and AUC for single risk factor in predicting OM in lung adenocarcinoma patients.

Factor	Cutoff value	Sensitivity (%)	Specificity (%)	AUC	*p*
CYFRA21-1 (ng/mL)	6.785	0.936	0.794	0.928	<0.001
CA-125 (U/mL)	66.295	0.745	0.744	0.749	<0.001
CA-153 (U/mL)	13.005	0.830	0.580	0.758	<0.001
CYFRA21-1+CA-125	—	0.814	0.851	0.890	<0.001
CYFRA21-1+CA-153	—	0.769	0.872	0.880	<0.001
CA-125+CA-153	—	0.682	0.809	0.809	<0.001
CYFRA21-1+CA-125+CA-153	—	0.807	0.851	0.888	<0.001

Sensitivity and specificity were obtained at the point of cutoff value. *p* < 0.05 represented statistical significance. AUC: area under the curve; CI: confidence interval; OM: ocular metastasis.

**Table 6:** The risk factors of metastases of lung cancer.

Author	Year	Histopathological type	Metastatic sites	Risk factor
Pollan [37]	2003	NSCLC	NS	CA-125
Oshiro [38]	2004	Adenocarcinoma	Liver	AFP
Cabreraalarcon [39]	2011	NS	NS	CYFRA21-1
Lee [30]	2012	NSCLC	Brain	CEA
Chen [31]	2015	NS	Lymph node	CYFRA21-1, CEA
Chen [40]	2015	NSCLC	Brain	NSE
Zhou [41]	2017	NS	Bone	CA-125, ALP
Morita [42]	2019	NSCLC	Intertrabecular vertebral	CEA

NS: not specific; NSCLC: non-small-cell lung cancer.

## Data Availability

All data is available on request.
